# Association of COVID-19 Severity with Comorbidities: Results from the World Trade Center Health Registry

**DOI:** 10.3390/ijerph23010010

**Published:** 2025-12-20

**Authors:** Janette Yung, Rebecca D. Kehm, Jiehui Li, James E. Cone

**Affiliations:** 1 World Trade Center Health Registry, Center for Population Health Data Science, New York City Department of Health and Mental Hygiene, Long Island City, NY 11101, USA; jyung@health.nyc.gov (J.Y.); jli3@health.nyc.gov (J.L.); 2 Department of Epidemiology, Mailman School of Public Health, Columbia University, New York, NY 10032, USA; rk2967@cumc.columbia.edu

**Keywords:** COVID-19 severity, hospitalization, World Trade Center disaster, pre-existing physical health condition

## Abstract

The impact of physical health conditions on coronavirus disease of 2019 (COVID-19) severity in World Trade Center disaster-exposed populations remains understudied. We examined the association of type, number and diagnosis time of pre-existing health conditions with COVID-19 severity, using the WTC Health Registry (WTCHR). We analyzed 3568 WTCHR enrollees with self-reported severe acute respiratory syndrome coronavirus 2 (SARS-CoV-2) infection in a 2021 follow-up survey. COVID-19 severity was measured by self-reported symptom duration (<2, 2–4, and >4 weeks) and hospitalization (hospitalized versus not). Pre-existing gastroesophageal reflux disease (GERD), respiratory conditions, cardiovascular conditions, and diabetes were self-reported and categorized into four groups (no diagnosis, post-9/11, pre-9/11, and undefinable). We used multinomial logistic regression and binary logistic regression to analyze the association of comorbidities with COVID-19 symptom duration and hospitalization, respectively, adjusting for post-traumatic stress disorder and demographic factors. Analysis was also conducted separately by enrollee type: rescue and recovery workers (RRW) vs. community members (non-RRW). Having all four health conditions post-9/11 was associated with longer symptom duration after SARS-CoV-2 infection (>4 weeks) among RRW (AOR: 2.66, 95% CI: 1.03–6.87). Reporting a post-9/11 respiratory condition was associated with an increased risk of being hospitalized among RRW and an increased risk of longer symptom duration (>4 weeks) among non-RRW. While post-9/11 diabetes was associated with an increased risk of longer symptom duration among RRW, post-9/11 GERD and pre-9/11 cardiovascular conditions were associated with an increased risk of longer symptom duration and being hospitalized among non-RRW, respectively. The impact of certain health conditions on COVID-19 severity varied across enrollee types and time of diagnosis. Given the lasting health impacts of 9/11-related exposures, targeted medical surveillance and proactive healthcare interventions are critical for mitigating the risk of severe COVID-19 illness in this population.

## 1. Introduction

The World Trade Center (WTC) attacks on 11 September 2001 killed thousands of people and exposed many more to hazardous materials resulting from the collapse of the Twin Towers and fires from the initial impact [1,2]. Previous studies reported an increased incidence of physical and mental health conditions after 9/11 among the WTC-exposed population compared to the general population. This includes an increased risk of asthma, chronic obstructive pulmonary disease (COPD), and other respiratory conditions; gastroesophageal reflux disease (GERD); cardiovascular conditions; diabetes; and post-traumatic stress disorder (PTSD) [3,4,5,6,7,8,9]. Given the high burden of mental and physical morbidities in the WTC-exposed population, it is likely that this population is more vulnerable to new and emerging health threats, such as the novel coronavirus disease 2019 (COVID-19), than the general population.

It has been reported that people with severe or multiple physical morbidities are more likely to experience severe or worse clinical outcomes following severe acute respiratory syndrome coronavirus 2 (SARS-CoV-2) infection [10,11]. Among the most common health conditions associated with severe COVID-19 illness are cardiovascular diseases and hypertension, diabetes, respiratory diseases, and GERD [10,11,12,13,14]. Moreover, a recent meta-analysis reported that previous use of a proton pump inhibitor, one of the most used drugs for gastric acid suppression, was associated with an increased risk of progression to severe COVID-19 [15].

The risks of physical health conditions associated with 9/11 exposure among exposed populations have been well-studied. Previous studies reported that longer duration and more intense exposure among the WTC-exposed rescue and recovery workers (RRW) are linked to cardiometabolic risk, cardiovascular disease and stroke [16,17,18,19], including higher incidence of diabetes, hypertension and worsening trajectories of glucose and systolic blood pressure over time [16]. Furthermore, the incidences of respiratory conditions such as asthma, chronic bronchitis, chronic obstructive pulmonary disease, airway hyperreactivity, and accelerated lung function decline are also increased among the WTC-exposed RRW compared to the general population and are strongly correlated with the intensity and duration of exposure to WTC dust and smoke [20]. These respiratory conditions are linked to metabolic syndrome, which is a known risk factor for cardiovascular disease. Similarly, among the community members (non-RRW), persistent upper and lower respiratory symptoms with lung function abnormalities were also reported for those who were exposed to pulverized dust, gas, and fumes after WTC destruction [21]. Furthermore, upper aerodigestive tract irritation such as GERD may potentially progress to major cardiovascular events and is associated with self-reported myocardial infarction among all of the WTC-exposed population [22]. Because of the heightened prevalence of these health conditions among the WTC-exposed population, which overlap with health conditions associated with severe SARS-CoV-2 infection outcome, people who were WTC-exposed may be at increased risk for severe COVID-19 illness after SARS-CoV-2 infection.

A previous cohort study of WTC rescue and recovery responders has already reported that those with pre-existing GERD, heart disease, and obstructive airway diseases were more likely to have severe COVID-19 illness than those without any of these conditions [23], while another study of Fire Department of New York responders who served in the WTC disaster reported that having a greater lung function decline rate prior to the pandemic was associated with severe COVID-19 illness [24]. No studies have yet examined the risk of severe COVID-19 outcome among WTC-exposed civilians. Given that WTC-exposed community members generally experienced different types of WTC exposure, risk of health conditions, and had very different demographic characteristics than RRW [21], it is hypothesized that the outcome of COVID-19 infection may differ between WTC-exposed RRW and non-RRW.

In this study, we conducted the first analysis of SARS-CoV-2 infection severity among enrollees in the World Trade Center Health Registry (WTCHR), which includes both RRW and non-RRW exposed to the WTC terrorist attacks. We examined whether the type, time of diagnosis relative to the WTC attacks, and number of post-9/11 physical health conditions in the WTC-exposed population were associated with the severity of COVID-19, as indicated by length of COVID-19 symptoms and related hospitalization in the 9/11-exposed population. We analyzed these associations in both the overall sample and separately for WTC RRW and non-RRW. This study did not collect data on Long COVID, which is addressed in a separate study.

## 2. Materials and Methods

### 2.1. Study Sample

The WTCHR was established in 2002 to conduct longitudinal follow-up on individuals who were exposed to the WTC attacks in New York City on 11 September 2001 (9/11). The methods for recruitment into the WTCHR are described in detail elsewhere [25,26]. In brief, the WTCHR completed enrollment in 2003–2004 and has recruited over 71,000 participants. The cohort in this study includes rescue/recovery workers, lower Manhattan residents, office workers, students, and passersby. All enrollees provided verbal informed consent at enrollment (Wave 1), followed by completion of a detailed questionnaire related to sociodemographic characteristics, WTC exposure, and physical and mental health history. Since enrollment, four subsequent waves of surveys were completed in 2006–2007 (Wave 2), 2011–2012 (Wave 3), 2015–2016 (Wave 4), and 2020–2021 (Wave 5). From April 2020 to February 2021, the Wave 5 survey was disseminated to nearly 40,000 eligible enrollees who completed the WTCHR’s Waves 1, 2, and at least one of the Waves 3 and 4 surveys.

In addition to five waves of survey data collected from the WTCHR cohort, the WTCHR has also conducted more focused, in-depth surveys to monitor and perform surveillance activities on newly emerging conditions. To capture individual-level experiences of the COVID-19 pandemic, a COVID-19 survey was developed and distributed to 36,831 eligible enrollees from January through August 2021. A total of 22,301 enrollees responded to the COVID-19 survey. A second WTCHR COVID-19 survey to address Long COVID, among other topics, was fielded between December 2022 and September 2023 among enrollees who had responded to the above COVID-19 survey, and this will be the source for future analyses.

**Figure 1:**
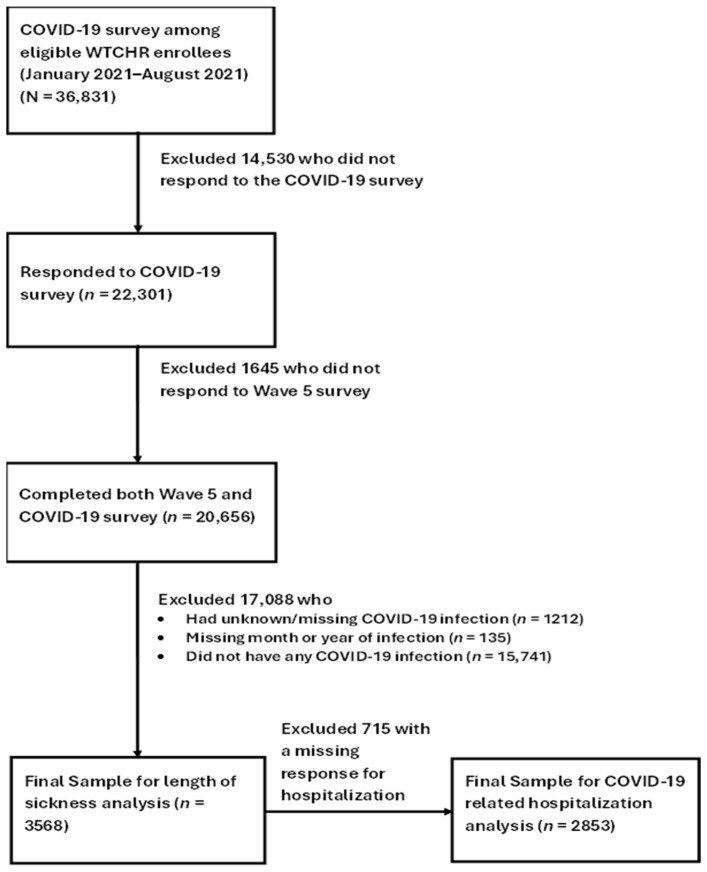
Flowchart of Sample Selection and Number of Enrollees in the Analyses.

The study sample of the present study consisted of those who reported having SARS-CoV-2 infection on the COVID-19 survey among enrollees who completed both the Wave 5 and the 2021 COVID-19 surveys (*n* = 20,656). We excluded enrollees who had unknown/missing self-reported COVID-19 infection (*n* = 1212), missing month or year of infection (*n* = 135) or reported that they did not have any COVID-19 infection (*n* = 15,741). The final sample for the analysis of symptom duration included 3568 enrollees. In the analyses involving COVID-19-related hospitalization, we further excluded 715 enrollees with missing data on hospitalization (final sample for hospitalization analysis *n* = 2853) (Figure 1).

### 2.2. Outcome Variables

Severity of SARS-CoV-2 infection was assessed by two variables: (1) duration of SARS-CoV-2 infection symptoms, and (2) hospitalization status. Duration was defined by the following question from the COVID-19 survey: “How long were you sick with COVID-19?”, which we categorized into a three-level variable (<2 weeks, between 2 and 4 weeks, and >4 weeks). We chose these cut-off points so that the reference level would conservatively capture mild COVID infections, which have an estimated median duration of approximately 10 days [27]. Hospitalization status was operationalized as a dichotomous variable (hospitalized vs. not hospitalized) derived from the survey question: “How long were you hospitalized for COVID-19 illness?” We did not ask for Long COVID status in the current survey, as this will be addressed in analyses of the WTCHR’s second COVID survey.

### 2.3. Exposures of Interest

The exposures of interest were self-reported physician-diagnosed pre-existing GERD, respiratory conditions (asthma, reactive airway dysfunction syndrome, and COPD), cardiovascular conditions (hypertension, angina, heart attack, coronary heart disease, and stroke), and diabetes. We chose these specific physical conditions because in addition to being the most common health conditions associated with severe COVID-19 illness, previous studies reported increased risks of these conditions among the WTC-exposed population [3,4,5,6,7,8,9]. Each of these physical conditions were grouped into 4 categories: (1) No diagnosis, (2) diagnosed post-9/11, (3) diagnosed pre-9/11, and (4) undefinable, based on responses from all primary wave surveys up until Wave 5. A common question regarding whether the enrollee has been told by a doctor or other health professional that he/she had the physical condition, followed by the year he/she was first told of the diagnosis, was asked in all Registry’s primary wave surveys. Responses from all surveys were aggregated and summarized based on step-wise logic rules, taking as precedent the most recent non-missing response over prior responses. Enrollees were considered as diagnosed post-9/11 if they consistently reported a year of diagnosis after 9/11, or if they reported no diagnosis in an earlier survey and later reported the condition with post-9/11 or unknown year of diagnosis, without ever reporting diagnosed pre-9/11. Enrollees were considered as diagnosed pre-9/11 if they reported the condition with a year of diagnosis pre-9/11, without ever reporting no diagnosis or year of diagnosis post-9/11. Undefinable included enrollees who reported no diagnosis, pre-9/11, and post-9/11 inconsistently across all surveys, except for those who reported no diagnosis in an earlier survey followed by diagnosed post-9/11 only. Based on their respective physiological systems, individual post-9/11 physical conditions were also examined as a summary variable of total number of types of post-9/11 physical conditions, ranging from 0 (no post-9/11 physical comorbidities across types reported) to 4 (all post-9/11 physical conditions across types reported).

### 2.4. Covariates

Covariates were chosen a priori and included in the analyses were age (<45, 45–64, and ≥65 years), gender (female, male), race/ethnicity (non-Hispanic White, non-Hispanic Black, Hispanic, non-Hispanic Asian, and non-Hispanic Other), marital status (now married/living with partner, divorced or separated, widowed, and never married), educational attainment (less than high school, high school/general education degree (GED), some college, and college graduate or above), household income (<$50K, $50K–<$75K, $75K–$150K, and $150K+), smoking history (never, former, and current), body mass index (BMI) (normal and underweight, overweight, and obese), and ever having probable WTC-related PTSD (Yes vs. No). Probable WTC-related PTSD symptoms were assessed using the 20-item scale version (PCL-5). Enrollees with a score > 33 were considered ever having probable PTSD. All covariates, except for gender, race/ethnicity (Wave 1), were obtained from either Wave 4 or Wave 5 based on the most recent report prior to the COVID infection. Enrollee type (rescue and recovery worker versus community member) was also determined at enrollment (Wave 1).

### 2.5. Data Analysis

We used multinomial logistic regression to estimate multivariable-adjusted odds ratios (AOR) for the associations of each of the four types of pre-existing physical conditions (GERD, diabetes, respiratory, and cardiovascular conditions) with duration of COVID-19-related illness, and binary logistic regression for the association with hospitalization, adjusting for covariates. We examined associations in overall sample and stratified by enrollee type (RRW versus non-RRW), which was self-reported at enrollment. All statistical analyses were performed using SAS v9.4 (Cary, NC, USA), with a two-sided significance level of ≤0.05. All procedures and the study were approved by the Institutional Review Board of the New York City Department of Health and Mental Hygiene.

## 3. Results

In the study sample of 3568 Registry enrollees who reported having had SARS-CoV-2 infection, 2038 (57.1%) reported being sick with COVID-19 for less than 2 weeks, 903 (25.3%) for 2 to 4 weeks, and 550 (15.4%) for more than four weeks (Table 1). Fifty-three percent of the sample were community members. About fifty-nine percent of the sample had at least one post-9/11 physical health condition prior to COVID-19 diagnosis. Over half of the sample had obtained a college degree or higher. A higher proportion of those who reported being sick for longer than 4 weeks versus less than 2 weeks (30.6% vs. 23.4%) or hospitalized versus not hospitalized for COVID-19 infection (42.2% vs. 24.2%) were 65 years or older. Among those who responded to the hospitalization question (*n* = 2853), 9.5% (*n* = 270) reported being hospitalized for COVID-19 infection. Compared to those who reported being sick for less than 2 weeks, those who reported being sick for longer than 4 weeks were more likely to be non-Hispanic Black and Hispanic (10.2% and 13.3% vs. 5.2% and 9.6%, respectively). Similarly, a higher proportion of those who reported being hospitalized for COVID-19 infection versus not hospitalized were non-Hispanic Black and Hispanic (11.9% and 12.2% vs. 6.1% and 11.0%, respectively). Moreover, a higher proportion of those who reported being sick for longer than 4 weeks versus less than 2 weeks or hospitalized for COVID-19 infection versus not hospitalized were obese, had post-9/11 diagnosis of GERD, a respiratory condition, cardiovascular disease, or diabetes.

Table 2 shows the adjusted odds ratios (AORs) and 95% confidence intervals (95% CI) for the associations of each comorbid physical conditions by time of diagnosis with length of sickness and hospitalization for COVID-19, respectively. Overall, having a post-9/11 diagnosis of GERD (AOR: 1.43, 95% CI: 1.13–1.82) or post-9/11 respiratory condition (AOR: 1.52, 95% CI: 1.17–1.98) was significantly associated with being sick for longer than 4 weeks versus less than 2 weeks (Table 2). Having a post-9/11 respiratory condition (AOR: 1.78, 95% CI: 1.27–2.49) or pre-9/11 cardiovascular condition (AOR: 2.37, 95% CI: 1.38–4.08) was associated with COVID-19 related hospitalization.

**Figure 2:**
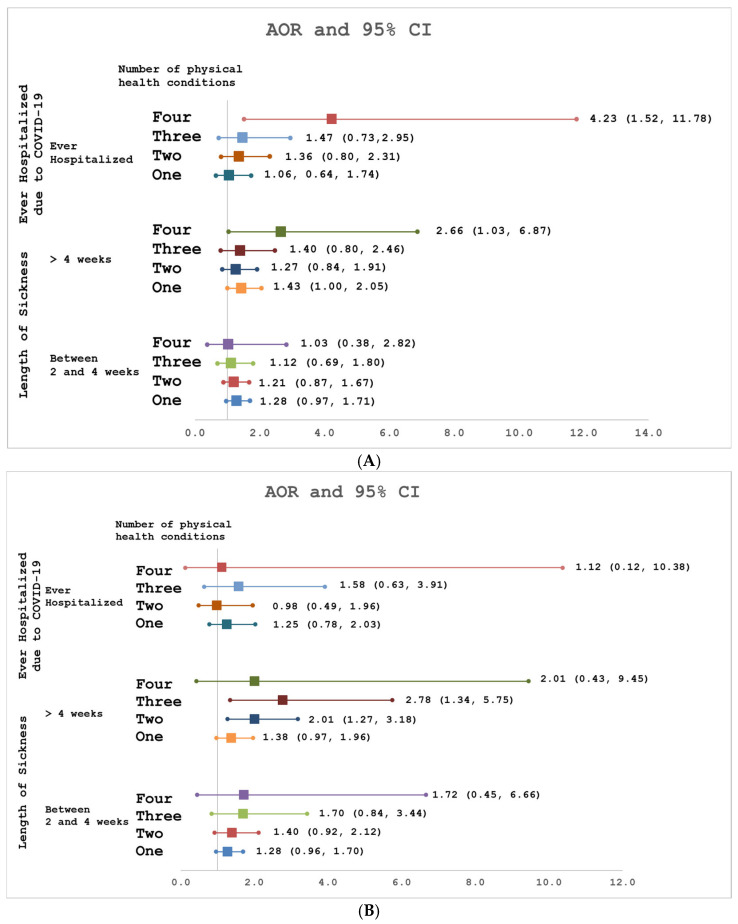
(**A**) Association of number of post-9/11 physical conditions with ever hospitalized for COVID and length of sickness among WTC Rescue and Recover Workers. (**B**) Association of number of post-9/11 physical conditions with ever hospitalized for COVID and length of sickness among WTC-exposed Community Members.

Among rescue and recovery workers, having post-9/11 diabetes (AOR: 1.57, 95% CI: 1.02–2.41) was associated with being sick with COVID-19 for >4 weeks compared to <2 weeks. Only having a post-9/11 respiratory condition (AOR: 2.16, 95% CI: 1.38–3.39) was associated with being hospitalized for COVD-19. Having all four physical conditions post-9/11 versus none was associated with being sick for >4 versus <2 weeks after SARS-CoV-2 infection (AOR: 2.66, 95% CI: 1.03–6.87) and being hospitalized for COVID-19 (AOR: 4.23, 95% CI: 1.52–11.78) (Figure 2A).

Among community members, having a post-9/11 respiratory condition (AOR: 2.04, 95% CI: 1.36–3.05) or GERD (AOR: 1.57, 95% CI: 1.08–2.28) was associated with being sick for >4 weeks compared to <2 weeks after SARS-CoV-2 infection. Having a pre-9/11 cardiovascular condition (AOR: 2.99, 95% CI: 1.40–6.43) was associated with being hospitalized for COVID-19. Having three (AOR: 2.78, 95% CI: 1.34–5.75) or two (AOR: 2.01, 95% CI: 1.27–3.18) post-9/11 physical conditions versus none was associated with being sick for >4 weeks compared to <2 weeks after SARS-CoV-2 infection (Figure 2B). However, having at least one post-9/11 physical condition versus none was not associated with COVID-19 hospitalization.

## 4. Discussion

Overall, our findings are consistent with research conducted on the general population [10,11,12,13,14]. The associations between certain pre-existing physical health conditions and COVID-19 severity that were reported in the general population were also seen among the WTC-exposed population. Specifically, we found that having a post-9/11 diagnosis of respiratory condition or GERD was associated with being sick for longer duration after SARS-CoV-2 infection among community members, while having a post-9/11 diagnosis of diabetes was associated with being sick for longer duration after SARS-CoV-2 infection among responders. Additionally, while having a post-9/11 respiratory condition was associated with being hospitalized for COVID-19 in responders, having a pre-9/11 cardiovascular condition was associated with being hospitalized for COVID-19 in community members. The likelihood of having a longer duration of sickness and being hospitalized for COVID-19 increased with the number of post-9/11 physical health conditions among RRW. These findings support the need to monitor and provide targeted healthcare for WTC-exposed individuals with pre-existing health conditions, as they often have a higher burden of such conditions compared to the general population and therefore face an increased risk of severe COVID-19 outcomes.

A systematic review of risk factors for severe clinical outcomes revealed that COPD was the most strongly predictive comorbidity for both severe disease and admission to ICU, while dyspnea (i.e., shortness of breath) was the only significant symptom predictive for both severe disease and ICU admission [13]. Respiratory conditions, along with cardiovascular conditions and diabetes, are also widely known to increase the odds of hospitalization after SARS-CoV-2 infection in the general population [10,11,12,13,14]. The mechanisms linking comorbidities to increased COVID-19 severity are not fully understood but may involve increased expression of the Angiotensin-Converting Enzyme 2 protein, the primary receptor that SARS-CoV-2 uses to enter human cells [28,29]. Additionally, compromised immune function, chronic systemic inflammation, and underlying organ dysfunction may play a role in exacerbating disease severity in individuals with pre-existing chronic health conditions [28]. Therefore, it is reasonable that a higher number of types of comorbidities that include these conditions is associated with more severe SARS-CoV-2 infection [11].

Among the WTC RRW, we found that having a post-9/11 respiratory condition was associated with a 116% increased risk of being hospitalized for COVID-19. A previous study of COVID-19 severity among WTC responders also reported that having an obstructive airway disease was significantly associated with severe SARS-CoV-2 infection, defined as either having severe symptoms or being admitted to a hospital [16]. A novel finding of this study is that post-9/11 diabetes was associated with longer symptom duration among rescue and recovery workers, which was not reported in Lhuillier’s study [23]. Having multiple physical health conditions is more common among WTC responders than in the general population [30] and, as shown in this study, can further increase the risk of severe COVID-19 illness. Our findings emphasize the importance of managing underlying conditions to not only reduce the risk of severe COVID-19 outcomes but also mitigate the risk of severe illness from similar infectious diseases.

This study also provides some of the first data on COVID-19 severity among community members exposed to the WTC terrorist attacks. Interestingly, we found that the same health condition can have a different impact on COVID-19 severity among WTC community members compared to rescue and recovery workers. For example, while post-9/11 diabetes was associated with a longer duration of COVID-19 symptoms in rescue and recovery workers, this association was not observed in community workers. Furthermore, among community members, having post-9/11 GERD and a pre-9/11 cardiovascular condition was associated with increased risk of longer symptom duration and increased risk of being hospitalized, respectively. These associations were not observed among rescue and recovery workers. The reasons for these differences are unclear but may be due to the well-documented differences in demographic statuses, WTC environmental and traumatic exposures, and healthcare access between WTC rescue and recovery workers and community members. For example, our finding on pre-9/11 CVD among community members may implicate the difference in chronicity of the condition between WTC responders and community members, as professional responders often have better cardiovascular health than the general population due to the physical criteria for entry into the occupation. Additionally, WTC community members are relatively more diverse in race/ethnicity, income, education background, and other sociodemographic statuses than rescue and recovery workers [21,30]. They were also exposed to lower levels of the initial dust cloud and lingering environmental contamination in the area. Additionally, the levels of access to healthcare services may differ between these enrollee groups. While the World Trade Center Health Program (WTCHP) provides medically necessary treatment and monitoring for WTC-related conditions at no cost for both Responders and Survivors who were impacted by the disaster for interdisciplinary illnesses, the established enrolment criteria are different for each of these two groups. Taken together, these may explain the difference in impact of COVID-19 severity by the same health condition between the groups. Given that this is the first study to identify these differences, further research is needed to better understand the underlying factors that influence COVID-19 severity across different enrollee groups.

Our study has several limitations. First, we cannot discount the possibility of response bias, as all data in this study are based on self-reported information. The mean number of months between time of COVID survey completion and the month and year of reported infection was 9 months, with a range of 0–19 months. Though it is unlikely for enrollees to forget an important lifetime event such as hospitalization, those who had the infection more recently would be more likely to recall the length of sickness accurately than having the infection earlier in the pandemic. Second, selection bias may have occurred because individuals with no or mild symptoms might have gone undetected and would thus have been excluded from this study. This issue may have been particularly pronounced early in the pandemic when testing was not widely available. Third, the COVID-19 survey was only offered to a subgroup of the WTCHR who had answered several earlier waves, further limiting the generalizability of this study and may skew the results in favor of those who were able to respond to the surveys. Fourth, although COVID-19 severity is associated with a wide range of new or worsened medical conditions across multiple body systems, we focused on physical health conditions that are most frequently documented and clinically impactful in this study. This allowed us to conduct a rigorous and interpretable analysis and to provide a clear foundation for future investigations. Our focuses on these major categories represent a first step, and future work can expand to additional conditions. Lastly, at the time of this study, data on long COVID that was collected in the second WTCHR COVID-19 survey were not readily available. Since having a more severe infection is a risk factor for long COVID [31,32], future studies incorporating these follow-up data to examine the association between acute severity and long COVID are warranted, to improve the understanding of the long-term impact of SARS-CoV-2 infection among WTC-exposed populations.

## 5. Conclusions

This study reinforces the existing evidence that comorbidities increase the risk of acute SARS-CoV-2 infection severity while also providing new insights into how other underlying factors may modify this relationship among the WTC-exposed population. Notably, we found that the impact of certain health conditions that were diagnosed after 9/11 on COVID-19 severity varied across different enrollee groups, suggesting that differences in demographics, 9/11-related exposures, and access to medical care may influence disease outcomes. These findings underscore the complex interplay between pre-existing health conditions and external factors, highlighting the need for targeted research and healthcare strategies to address the unique risks faced by different populations. Moreover, this study adds to the existing literature by illuminating the indirect impact of WTC exposure on health through heightening of disease severity in the context of a new and emerging health threat, expanding beyond the direct long-term impact of WTC exposure on the development of chronic health conditions. This further underlines the importance of continued monitoring and care of WTC-exposed individuals to prevent further deterioration of health.

## Figures and Tables

**Table 1:** Characteristics of Study Sample by COVID-19 Severity.

		Length of Sickness, Weeks			COVID19-Related Hospitalization ^†^	
Characteristics	All * n (%)	<2 *n* (%)	2–4 *n* (%)	>4 *n* (%)	No *n* (%)	Yes *n* (%)
Total	3568	2038	903	550	2583	270
Age at Wave 5, years						
<45	326 (9.14)	211 (10.35)	76 (8.42)	32 (5.82)	240 (9.29)	8 (2.96)
45 to 64	2318 (64.97)	1350 (66.24)	574 (63.57)	350 (63.64)	1718 (66.51)	148 (54.81)
65+	924 (25.90)	477 (23.41)	253 (28.02)	168 (30.55)	625 (24.20)	114 (42.22)
Gender						
Female	1261 (35.34)	701 (34.40)	313 (34.66)	212 (38.55)	874 (33.84)	73 (27.04)
Male	2307 (64.66)	1337 (65.60)	590 (65.34)	338 (61.45)	1709 (66.16)	197 (72.96)
Race/Ethnicity						
Non-Hispanic White	2712 (76.01)	1621 (79.54)	652 (72.20)	385 (70.00)	1997 (77.31)	182 (67.41)
Non-Hispanic Black	243 (6.81)	106 (5.20)	77 (8.53)	56 (10.18)	158 (6.12)	32 (11.85)
Hispanic	401 (11.24)	196 (9.62)	122 (13.51)	73 (13.27)	285 (11.03)	33 (12.22)
Asian	114 (3.20)	63 (3.09)	31 (3.43)	17 (3.09)	80 (3.10)	13 (4.81)
All other	98 (2.75)	52 (2.55)	21 (2.33)	19 (3.45)	63 (2.44)	10 (3.70)
Enrollee type						
Rescue/Recovery workers	1675 (46.95)	942 (46.22)	433 (47.95)	265 (48.18)	1200 (46.46)	121 (44.81)
Community members	1893 (53.05)	1096 (53.78)	470 (52.05)	285 (51.82)	1383 (53.54)	149 (55.19)
Marital status						
Married/living with partner	2453 (72.88)	1426 (74.39)	621 (72.38)	365 (69.79)	1816 (74.37)	188 (72.31)
Divorced/separated	413 (12.27)	217 (11.32)	118 (13.75)	62 (11.85)	282 (11.55)	29 (11.15)
Widowed	98 (2.91)	52 (2.71)	29 (3.38)	14 (2.68)	67 (2.74)	11 (4.23)
Never married	402 (11.94)	222 (11.58)	90 (10.49)	82 (15.68)	277 (11.34)	32 (12.31)
Educational attainment						
High School/GED or lower	481 (14.32)	245 (12.78)	143 (16.74)	78 (15.06)	342 (14.05)	57 (21.92)
Some College	1022 (30.43)	545 (28.43)	272 (31.85)	176 (33.98)	755 (31.02)	88 (33.85)
College graduate or above	1855 (55.24)	1127 (58.79)	439 (51.41)	264 (50.97)	1337 (54.93)	115 (44.23)
Household income						
<$50,000	510 (15.73)	249 (13.44)	139 (16.73)	100 (20.12)	330 (14.04)	48 (19.51)
$50K–$74,999	375 (11.57)	196 (10.58)	97 (11.67)	72 (14.49)	266 (11.31)	33 (13.41)
$75K–$149,999	1262 (38.93)	714 (38.55)	330 (39.71)	197 (39.64)	924 (39.30)	105 (42.68)
150K+	1095 (33.78)	693 (37.42)	265 (31.89)	128 (25.75)	831 (35.35)	60 (24.39)
Smoking history						
Never smoker	2100 (63.39)	1187 (62.51)	540 (64.52)	341 (66.99)	1558 (64.81)	149 (59.13)
Former smoker	1005 (30.34)	594 (31.28)	247 (29.51)	137 (26.92)	702 (29.20)	87 (34.52)
Current smoker	208 (6.28)	118 (6.21)	50 (5.97)	31 (6.09)	144 (5.99)	16 (6.35)
BMI						
Normal and Underweight	764 (23.37)	474 (25.50)	177 (21.12)	96 (19.01)	541 (22.88)	36 (14.12)
Overweight	1294 (39.58)	761 (40.94)	325 (38.78)	186 (36.83)	972 (41.12)	89 (34.90)
Obesity	1211 (37.04)	624 (33.57)	336 (40.10)	223 (44.16)	851 (36.00)	130 (50.98)
Last known probable PTSD						
No	3111 (87.22)	1823 (89.49)	788 (87.26)	446 (81.09)	228 (84.44)	2284 (88.39)
Yes	456 (12.78)	214 (10.51)	115 (12.74)	104 (18.91)	42 (15.56)	300 (11.61)
Respiratory conditions (Asthma, RADS, Chronic Bronchitis, Emphysema or COPD)						
No diagnosis	1887 (54.10)	1175 (57.71)	486 (53.88)	226 (41.09)	1456 (56.39)	118 (43.70)
Post-9/11	789 (22.62)	428 (21.02)	203 (22.51)	158 (28.73)	527 (20.41)	86 (31.85)
Pre-9/11	178 (5.10)	102 (5.01)	44 (4.88)	32 (5.82)	132 (5.11)	15 (5.56)
Undefinable	634 (18.18)	331 (16.26)	169 (18.74)	134 (24.36)	467 (18.09)	51 (18.89)
Cardiovascular conditions (Hypertension, angina, heart attack, stroke, or CHD)						
No diagnosis	1587 (45.45)	966 (47.40)	400 (44.25)	221 (40.18)	1231 (47.64)	73 (27.04)
Post-9/11	1108 (31.73)	600 (29.44)	306 (33.85)	202 (36.73)	803 (31.08)	103 (38.15)
Pre-9/11	180 (5.15)	104 (5.10)	49 (5.42)	27 (4.91)	116 (4.49)	30 (11.11)
Undefinable	617 (17.67)	368 (18.06)	149 (16.48)	100 (18.18)	434 (16.80)	64 (23.70)
Diabetes						
No diagnosis	2928 (83.85)	1755 (86.11)	746 (82.52)	427 (77.64)	2186 (84.60)	193 (71.48)
Post-9/11	343 (9.82)	170 (8.34)	94 (10.40)	79 (14.36)	245 (9.48)	44 (16.30)
Pre-9/11	46 (1.32)	20 (0.98)	15 (1.66)	11 (2.00)	30 (1.16)	11 (4.07)
Undefinable	175 (5.01)	93 (4.56)	49 (5.42)	33 (6.00)	123 (4.76)	22 (8.15)
Post-9/11 Gastroesophageal reflux disease (GERD)						
No diagnosis	2057 (58.96)	1260 (61.89)	516 (57.14)	281 (51.09)	1562 (60.50)	145 (53.70)
Post-9/11	983 (28.17)	532 (26.13)	261 (28.90)	190 (34.55)	709 (27.46)	84 (31.11)
Pre-9/11	54 (1.55)	26 (1.28)	15 (1.66)	13 (2.36)	42 (1.63)	4 (1.48)
Undefinable	395 (11.32)	218 (10.71)	111 (12.29)	66 (12.00)	269 (10.42)	37 (13.70)
Number of types of post-9/11 physical health co-morbidity						
None	1435 (41.15)	916 (44.99)	344 (38.18)	175 (31.82)	1102 (42.70)	84 (31.11)
One	1192 (34.18)	662 (32.51)	333 (36.96)	197 (35.82)	890 (34.48)	97 (35.93)
Two	602 (17.26)	328 (16.11)	159 (17.65)	115 (20.91)	407 (15.77)	55 (20.37)
Three	212 (6.08)	108 (5.30)	54 (5.99)	50 (9.09)	154 (5.97)	26 (9.63)
Four	46 (1.32)	22 (1.08)	11 (1.22)	13 (2.36)	28 (1.08)	8 (2.96)

* Some may not add up to the total sum due to missing. ^†^ Excluded missing (n = 715).

**Table 2:** Adjusted Odds Ratios (AOR) and 95% Confidence Intervals (CI) for the Association Between Each Pre-existing Condition and COVID-19 Severity, stratified by time of diagnosis *.

	Length of Sickness * (Referent: <2 Weeks)		COVID19-Related Hospitalization (Referent: Not Hospitalized)
Self-Reported Pre-Existing Health Condition	2–4 AOR (95% CI)	>4 AOR (95% CI)	AOR (95% CI)
**All ^†^**			
Any respiratory conditions (Asthma, RADS, Chronic Bronchitis, Emphysema or COPD)			
Post-9/11	1.08 (0.86, 1.35)	1.52 (1.17, 1.98)	1.78 (1.27, 2.49)
Pre-9/11	1.05 (0.71, 1.56)	1.22 (0.76, 1.96)	1.68 (0.92, 3.08)
Undefinable	1.20 (0.95, 1.51)	1.77 (1.35, 2.32)	1.14 (0.78, 1.69)
Any selected cardiovascular conditions (Hypertension, angina, heart attack, stroke, or CHD)			
Post-9/11	1.06 (0.86–1.30)	1.17 (0.91–1.51)	1.37 (0.95, 1.98)
Pre-9/11	0.90 (0.60, 1.36)	0.75 (0.45, 1.28)	2.37 (1.38, 4.08)
Undefinable	0.71 (0.55, 0.91)	0.85 (0.63, 1.16)	1.69 (1.13, 2.52)
Diabetes			
Post-9/11	1.04 (0.78, 1.40)	1.37 (0.99, 1.91)	1.40 (0.94, 2.09)
Pre-9/11	1.64 (0.79, 3.39)	1.76 (0.77, 4.04)	2.03 (0.93, 4.44)
Undefinable	0.93 (0.63, 1.38)	1.14 (0.73, 1.76)	1.32 (0.77, 2.26)
Gastroesophageal reflux disease (GERD)			
Post-9/11	1.27 (1.04, 1.55)	1.43 (1.13, 1.82)	1.25 (0.90, 1.72)
Pre-9/11	1.45 (0.73, 2.85)	1.20 (0.52, 2.78)	0.94 (0.32, 2.76)
Undefinable	1.20 (0.91, 1.57)	1.17 (0.84, 1.64)	1.06 (0.68, 1.65)
**WTC rescue/recovery workers**			
Any respiratory conditions (Asthma, RADS, Chronic Bronchitis, Emphysema or COPD)			
Post-9/11	0.85 (0.63, 1.14)	1.24 (0.87, 1.78)	2.16 (1.38, 3.39)
Pre-9/11	1.17 (0.65, 2.09)	0.74 (0.30, 1.83)	1.34 (0.45, 4.01)
Undefinable	1.11 (0.80, 1.54)	1.92 (1.32, 2.79)	1.71 (1.02, 2.87)
Any selected cardiovascular conditions (Hypertension, angina, heart attack, stroke, or CHD)	0.96 (0.75–1.24)	0.98 (0.71–1.35)	
Post-9/11	1.07 (0.81, 1.42)	1.05 (0.74, 1.49)	1.22 (0.75, 1.98)
Pre-9/11	1.15 (0.63, 2.09)	1.19 (0.58, 2.43)	1.99 (0.90, 4.42)
Undefinable	0.73 (0.51, 1.04)	0.82 (0.53, 1.25)	1.65 (0.95, 2.85)
Diabetes			
Post-9/11	1.08 (0.73, 1.58)	1.57 (1.02, 2.41)	1.35 (0.80, 2.28)
Pre-9/11	1.94 (0.70, 5.33)	1.87 (0.54, 6.49)	2.17 (0.72, 6.61)
Undefinable	0.69 (0.38, 1.24)	1.49 (0.83, 2.67)	0.81 (0.33, 1.98)
Gastroesophageal reflux disease (GERD)			
Post-9/11	1.20 (0.93, 1.55)	1.36 (0.99, 1.55)	1.42 (0.94, 2.14)
Pre-9/11	1.98 (0.71, 5.53)	0.85 (0.18, 4.10)	0.68 (0.08, 5.57)
Undefinable	0.92 (0.61, 1.38)	1.09 (0.67, 0.78)	0.79 (0.39, 1.62)
**Community members**			
Any respiratory conditions (Asthma, RADS, Chronic Bronchitis, Emphysema or COPD)			
Post-9/11	1.53 (1.08, 2.18)	2.04 (1.36, 3.05)	1.27 (0.74, 2.19)
Pre-9/11	1.03 (0.60, 1.74)	1.52 (0.85, 2.73)	1.72 (0.82, 3.63)
Undefinable	1.34 (0.97, 1.87)	1.68 (1.13, 2.49)	0.72 (0.39, 1.33)
Any selected cardiovascular conditions (Hypertension, angina, heart attack, stroke, or CHD)			
Post-9/11	1.06 (0.77, 1.46)	1.36 (0.93, 1.97)	1.59 (0.91, 2.79)
Pre-9/11	0.76 (0.44, 1.33)	0.48 (0.22, 1.07)	2.99 (1.40, 6.43)
Undefinable	0.69 (0.47, 1.00)	0.90 (0.58, 1.40)	1.67 (0.91, 3.07)
Diabetes			
Post-9/11	0.99 (0.62, 1.57)	1.16 (0.69, 1.93)	1.35 (0.72, 2.53)
Pre-9/11	1.41 (0.49, 4.07)	1.73 (0.55, 5.41)	2.04 (0.65, 6.36)
Undefinable	1.23 (0.72, 2.09)	0.83 (0.42, 1.64)	2.04 (0.99, 4.21)
Gastroesophageal reflux disease (GERD)			
Post-9/11	1.36 (0.98, 1.90)	1.57 (1.08, 2.28)	0.96 (0.55, 1.68)
Pre-9/11	1.18 (0.48, 2.94)	1.44 (0.52, 3.96)	1.16 (0.32, 4.23)
Undefinable	1.51 (1.04, 2.20)	1.31 (0.82, 2.10)	1.39 (0.78, 2.49)

Abbreviation: RADS = Reactive airway dysfunction syndrome, COPD = Chronic obstructive pulmonary disease, CHD = coronary heart disease. * Adjusted for age, gender, race/ethnicity, education attainment, household income, smoking history, BMI (body mass index) and PTSD. ^†^ Also included enrollee type in multivariable analysis.

## Data Availability

World Trade Center Health Registry data presented in this study are available on request from the corresponding author following review of applications to the Registry from external researchers. The data are not publicly available due to privacy or ethical restrictions.

## Abbreviations

The following abbreviations are used in this manuscript:

AOR	Adjusted odds ratio
BMI	Body mass index
CI	Confidence interval
COPD	Chronic obstructive pulmonary disease
GED	General education degree
GERD	Gastroesophageal reflux disease
PTSD	Post-traumatic stress disorder
WTC	World Trade Center
WTCHR	World Trade Center Health Registry
